# OLDER ADULTS LEAVING THE WORKFORCE: SENSORY LOSS, RETIREMENT, AND DISABILITY

**DOI:** 10.1093/geroni/igac059.2504

**Published:** 2022-12-20

**Authors:** Emmanuel Garcia Morales, Lama Assi, Nicholas Reed

**Affiliations:** Cochlear Center for Hearing and Public Health, Baltimore, Maryland, United States; Louisiana State University Health Center, New Orleans, Louisiana, United States; Johns Hopkins Bloomberg School of Public Health, Baltimore, Maryland, United States

## Abstract

Hearing loss (HL), vision loss (VL), and their combination (dual sensory loss, DSL) are common among older adults. Sensory loss impacts labor productivity which might result in departures from the workforce. Whether older adults leave due to retirement or a disability, and how these responses are associated with sensory loss remains unexplored. Using the 2004-2018 rounds of the Health and Retirement Study, self-reported sensory loss (No Impairment/HL/VL/DSL) at baseline, and reason for leaving the workforce (retirement or disability) were observed. Competing risk models models for departures from the workforce treating retirement or disability as a competing risk were estimated. Among 5,201 adults employed at baseline, ages 50-94, 3,436 reported retirement, and 4254 reported a disability as a reason for not working. In Fine-Gray models, treating retirement as a competing risk and adjusting for sociodemographic and clinical characteristics, we found that compared to older adults without impairments, DSL was associated with a 50% increase in the rate of departures from the labor force due to disability among adults in the in the risk group (SHR=1.51; 95% CI=1.09,2.11). In contrast, when treating disability as the competing risk, HL was associated with a 22% increase in the rate of departures labor force due to retirement (SHR=1.22; 95% CI=1.10,1.36) among adults in the risk group when compared to those without impairments. In sample of older adults, we provide evidence that the presence of sensory impairments is associated with departures from the workforce. Our results highlight differences in the type of departures by sensory loss.

